# A Psychosocial Analysis in a Context of Seismic and Volcanic Emergency: Well‐Being in the Campi Flegrei Area

**DOI:** 10.1002/jcop.70125

**Published:** 2026-07-08

**Authors:** Francesca Mazzella, Miriam Capasso, Daniela Caso

**Affiliations:** ^1^ Department of Humanities University of Naples Federico II Naples Italy

**Keywords:** Campi Flegrei, coping strategies, natural hazards, post‐traumatic stress symptoms, psycho‐social well‐being, trust, Volcanic risk perception

## Abstract

The Campi Flegrei area, one of the most active volcanic regions in Italy, has faced a prolonged crisis, characterized by recurring seismic events. By adopting a psychosocial and community‐based perspective, this study investigates how seismic and volcanic risk perception, perceived severity, trust in institutions and media, and coping strategies influence the psychological impact of the emergency. Additionally, residents' psychosocial well‐being was assessed using Keyes' Mental Health Continuum Model, classifying participants as flourishing, moderate, or languishing. Data were collected through a questionnaire administered to 532 residents living in the Campi Flegrei red zone. Regression analyses showed that risk perception, perceived severity, and maladaptive coping strategies positively predicted psychological impact, while trust in sources negatively predicted it. ANOVA showed that languishing individuals reported worse psychological outcomes. These results highlight the urgent need for targeted community‐based psychosocial interventions to mitigate the adverse effects of prolonged exposure to seismic and volcanic hazards.

## 1 Introduction

Active volcanoes pose significant, long‐term risks to nearby populations. Globally, over 500 million people live and work in the shadow of active volcanoes, often choosing to remain due to cultural ties, economic opportunities, or lack of alternatives. These communities face not only the immediate danger of an eruption but also the continual stress of volcanic unrest. Large calderas, for example, can experience repeated episodes of seismic and deformational activity over years or decades without erupting (Kilburn et al. 2023). Such chronic uncertainty can gradually wear on residents' psyches; periods of false alarms and non‐eruptive crises may even foster public complacency or mistrust toward future warnings (Kilburn et al. 2023).

Natural disasters, in general, are well known to take a toll on mental health. A substantial body of research has documented the psychological impacts of catastrophic events like earthquakes, hurricanes, and volcanic eruptions. The literature highlights that natural disasters can have a significant traumatic impact on psychosocial well‐being, often causing acute and chronic stress symptoms that may persist for months or even years (Neria et al. 2008; Norris et al. 2002). It is estimated that approximately 33% of individuals directly exposed to natural disasters will face mental health challenges, including post‐traumatic stress disorder (PTSD), anxiety, and depression (Norris et al. 2002; North and Pfefferbaum 2013). While the majority of individuals who experience a traumatic event due to natural disasters do not develop psychopathology, such calamities can threaten their psychosocial well‐being in many ways, and they can result in both short and long‐term psychological distress and thus create a significant burden of mental health conditions on individuals and the community affected by them (Saeed and Gargano 2022).

However, most research to date has focused on post‐disaster intervention and recovery (Kar 2009), whereas far less is known about the psychosocial impact of living under an ongoing threat. As noted in recent reviews, the vast majority of psychological studies and interventions are conducted after a disaster has occurred (Raccanello et al. 2023). Living with perpetual risk, such as next to an active volcano, means that risk becomes a daily condition that people must learn to manage and adapt (López‐Vázquez 2009). In line with a pre‐disaster perspective, comparative evidence from Vesuvius and Etna (Barberi et al. 2008; Carlino et al. 2008), Merapi (Dove 2008), and Popocatépetl (López‐Vázquez 2009) suggests that residents' responses to volcanic unrest are shaped by risk perception, trust, and everyday coping well before an eruption occurs. Cultural interpretations of risk, confidence in emergency plans, and the quality of risk communication can all influence protective intentions. Around Popocatépetl, for example, residents reported persistent feelings of insecurity and stress associated with volcanic activity while gradually adapting their daily lives to the omnipresent threat (López‐Vázquez 2009).

Such scenarios underscore the need for research on pre‐disaster psychosocial effects and how chronic hazard exposure shapes mental health, risk perceptions, and coping behavior before any catastrophic eruption or event. Despite growing attention to disaster mental health, comparatively little is known about how psychosocial functioning develops under conditions of prolonged exposure to volcanic unrest, particularly in contexts characterized by chronic uncertainty and recurrent episodes of activity. A broader international perspective is also needed, as insights from one volcanic region can inform risk management and community support strategies in other similar contexts. Understanding how people adapt in anticipation of hazardous events, and how such adaptation may influence responses when disasters occur, is essential for developing effective support systems. Indeed, disaster mental health experts emphasize that promoting resilience and preparedness before a calamity strikes can buffer adverse outcomes when a crisis occurs (Raccanello et al. 2023). This work addresses a clear gap in the hazard literature and offers practical implications for disaster preparedness in volcanically active regions worldwide.

These considerations suggest that psychosocial adaptation to volcanic unrest should be examined not only at the individual level but also within the broader community context. As scientific literature suggests, the well‐being of individuals cannot be separated from the health of the community as a whole (Sarason 1974). Thus, community resilience, the ability of communities to adapt to change and recover from crises, must be promoted alongside individual psychological health. In line with this perspective, the present study examines the role of both individual variables, such as coping strategies and perceived risk, and community‐level variables, such as trust in institutions and media in influencing the impact of the event.

To systematically capture this interplay, we rely on Keyes's Mental Health Continuum (MHC) as an overarching framework. By classifying respondents as flourishing, moderately healthy, or languishing (Keyes 2002), we establish distinct population‐level well‐being profiles. This classification allows us to move beyond treating risk perception, institutional trust, and coping strategies as isolated predictors of distress; instead, we can systematically link them to these profiles, integrating them into a comprehensive assessment of psychosocial functioning under prolonged uncertainty. In short, our objective is twofold: (1) to examine whether these psychosocial variables are associated with the ongoing impact of an event experienced under chronic unrest; and (2) to assess how the same variables differ across MHC categories, thereby linking everyday life under threat to population‐level well‐being profiles.

## 2 The Campi Flegrei Context

The volcanic area of Campi Flegrei is one of the most critical areas within the metropolitan city of Naples, in Southern Italy, due to its multi‐hazard features, high population density, and rich historical, archeological, and natural heritage. It is the largest volcanic caldera in Europe, partly emerged and partly submerged, and one of the most densely inhabited all over the world (Kilburn et al. 2023). The area is also characterized by the so‐called bradyseism, a cyclic slow ground deformation leading to the sinking of the ground level during certain periods followed by uplift phases (Lima et al. 2009).

The volcano has been restless since 1950. It last erupted in 1538 after an interval of about 3000 years; previous intervals have been as short as decades or centuries (Smith et al. 2011), so that a return to eruption after nearly 500 years is considered a realistic possibility (Kilburn et al. 2023). During the twentieth century, the area experienced two important bradyseismic crises (1970–1972 and 1982–1984). The latest one, which started in 2005 and is still ongoing, intensified over the past 2 years, with several shallow earthquakes of short duration and low intensity that largely contributed to raising the concern of citizens and local authorities (Galderisi and Limongi 2024).

The ongoing seismic activity in Campi Flegrei places a continuous psychological burden on the local population, fueled by high perceived risk, uncertainty about the future, and the awareness of living atop an active “supervolcano” (Longo 2018). This persistent tension contributes to elevated stress, anxiety, and vulnerability, impacting both individual well‐being and community dynamics. Consistent with this, Caffieri et al. (2025) provided empirical evidence of pre‐traumatic stress responses in residents of the Campi Flegrei area, showing that continuous bradyseismic activity and perceived environmental hazard can activate stress mechanisms typically associated with post‐disaster conditions. In short, Campi Flegrei's situation illustrates how intertwined geological processes and psychosocial dynamics become in a long‐term “slow disaster” scenario. Any effective risk reduction strategy here must therefore address not only the physical hazards but also the perceptions, fears, and coping capacities of the people who live with those hazards every day.

## 3 Theoretical Framework

### 3.1 Psychosocial Well‐Being and Psychosocial Factors under Chronic Seismic‐Volcanic Threat

The persistent uncertainty of the Campi Flegrei emergency significantly challenges residents' threat appraisal and coping capacities (Fearnley et al. 2012; Ricci et al. 2013). To interpret psychosocial functioning under these pre‐disaster conditions, the Mental Health Continuum (MHC; Keyes 2002) provides a useful organizational framework for understanding mental health as a multidimensional condition involving emotional, psychological, and social well‐being.

The MHC conceptualizes mental health as a continuum ranging from “flourishing,” characterized by high levels of emotional, psychological, and social well‐being, to “languishing,” marked by low well‐being and psychological distress. This model provides a multidimensional understanding of mental health that goes beyond the absence of mental illness, highlighting positive functioning and adaptation across emotional, psychological, and social domains (Keyes 2002, 2005). Within this framework, emotional well‐being refers to the presence of positive emotions and life satisfaction; psychological well‐being involves positive functioning at the individual level, including autonomy, personal growth and purpose in life (Ryff 1989); while social well‐being reflects an individual's sense of belonging, social integration, and contribution to society (Keyes 2005). This integrative perspective is consistent with Westerhof and Keyes' (2010) emphasis on considering emotional, psychological, and social well‐being jointly as interrelated components of a complete state of mental health. It also aligns with the World Health Organization's definition of mental health as a state of well‐being in which individuals can realize their abilities, cope with life stressors, work productively, and contribute to their community (World Health Organization 2004).

In chronic threat contexts such as Campi Flegrei, the MHC framework is especially relevant because prolonged uncertainty, repeated seismic activity, and perceived limitations in institutional communication and preparedness may progressively challenge all three dimensions of well‐being. Emotional well‐being may be particularly fragile when perceived danger reduces feelings of safety, calm, and life satisfaction. Psychological well‐being may be undermined by the disempowerment and unpredictability that accompany long‐term exposure to seismic and volcanic risk. The social dimension of well‐being may also be affected when residents feel isolated, unsupported, or mistrustful of institutions and information sources.

Previous applications of the MHC in the Italian setting have shown its usefulness for analyzing population well‐being and identifying distinct mental‐health profiles associated with different coping strategies and stress levels (e.g., Capone et al. 2020; Capone et al. 2014).

Building on this framework, we can classify respondents into three mental‐health profiles (flourishing, moderate, languishing) and test whether these categories systematically differ in risk perception, trust in institutions and media, coping strategies and impact of the event, thus providing theory‐driven leverage for targeted interventions. This alignment allows hypotheses to be specified at two levels (individual and population) while using a single, coherent framework to interpret emotional, psychological, and social functioning.

Within this framework, the variables included in the present study were selected because they capture complementary psychosocial processes through which chronic seismic‐volcanic threat may affect emotional, psychological, and social well‐being. Risk perception and perceived severity represent threat‐appraisal processes, closely related to emotional well‐being insofar as perceived danger may reduce feelings of safety, calm, and life satisfaction, and to psychological well‐being insofar as adaptation to a hazardous environment requires interpreting environmental cues and managing uncertainty (Keyes 2002, 2005). Coping strategies represent individual self‐regulatory responses linked to psychological functioning, particularly environmental mastery, adaptation to adversity, and preservation of a sense of control (Keyes 2005). Finally, trust in institutions and media represents an individual perception of the broader socio‐institutional context and is linked to social well‐being, because it shapes whether the social environment is perceived as coherent, reliable, and supportive (Keyes 1998, 2005). These variables were therefore not selected as isolated predictors, but as theoretically central components connecting individual threat appraisal, individual coping regulation, and the perceived socio‐institutional context within the MHC framework.

### 3.2 Risk Perception, Trust, and Coping under Chronic Threat

Operating at the emotional and threat‐appraisal level, risk perception is one of several important factors that can influence people's recognition of their vulnerability to volcanic hazards and their possible reactions (Ricci et al. 2013; Chester et al. 2002; Dibben and Chester 1999). Risk perception varies depending on individuals' impressions of potential direct harm to their lives and property, as well as whether they consider themselves involved or uninvolved in the threat. This, in turn, affects stress levels, and, consequently, the perceived impact of the event, as well as coping responses to a threatening situation (López‐Vázquez and Marván 2003). In other words, risk perception and personal involvement underpin the sense of vulnerability experienced by individuals, but they are also linked to the strategies people develop to cope with such threats.

This perceived vulnerability is inextricably linked to the social environment and the quality of risk communication. The bradyseismic crises that have struck the Campi Flegrei area over recent decades have contributed to widespread social vulnerability. This vulnerability is further aggravated by unclear communication strategies and the challenges of implementing effective emergency plans (Crescimbene et al. 2015; Longo 2018). Since most people form their perception of risk through information received from external sources (Lavanco 2003), the role of institutions, such as local and national governments, civil protection agencies, the scientific community, and the media becomes crucial in shaping public understanding of risk and influencing behavioral responses. Clear and consistent communication from these sources can enhance preparedness and facilitate effective emergency management, whereas conflicting or alarmist messages may lead to confusion, distrust, and irrational reactions (Lavanco 2003; Paton et al. 2000; Terpstra 2011). Trust, a key component of social capital, plays a deep role in this process. Confidence in institutions greatly influences how individuals view and react to risk, as it provides a sense of security and confidence in the management of hazardous situations (Siegrist and Cvetkovich 2000). Moreover, research suggests that the impact of disasters is not just about how people assess the threat but also how much they trust those in charge of responding to the crisis. Bonfanti et al. (2024) found that individuals with greater trust in institutional authorities experienced reduced stress during and after disasters, indicating that trust contributes to lessening the overall impact of such events. When people have confidence in the institutions managing the situation, they are less likely to feel overwhelmed or helpless. This highlights the critical role of institutional trust, not only in shaping risk perceptions but also in mitigating the emotional and psychological consequences of disasters, ultimately helping communities cope more effectively with these challenges.

Faced with this persistent perceived threat and the complexities of the socio‐institutional environment, the psychosocial impact of the Campi Flegrei emergency ultimately depends on individual coping strategies. Emotional reactions, as suggested by Loewenstein et al. (2001), are immediate and functional responses to danger. However, they can also complicate decision‐making during emergencies, as high‐pressure situations often lead to cognitive biases, reliance on heuristics, and reduced selective attention (Pietrantoni and Prati 2009). To cope with the uncertainty associated with seismic and volcanic risks, individuals develop strategies that help maintain physical and psychological stability. Studies on disaster events have shown that adaptive coping strategies can significantly reduce the psychological impact of the event and promote emotional and psychological recovery (Lindell and Perry 2012). In contrast, avoidance or denial of the threat is often associated with higher levels of psychological distress and can interfere with the ability to make effective decisions during crises (Coifman et al. 2007). The present study specifically focuses on two key coping mechanisms: denial coping and support‐seeking coping. Denial coping involves efforts to ignore or distance oneself from the stressful event and the emotions associated with it, often through distraction or social disengagement (Carver et al. 1989). On the other hand, support‐seeking coping, a form of emotion‐focused coping, entails seeking social support for emotional comfort and assistance. Social support has been widely recognized as a protective factor that can mitigate the effects of stress and promote better psychological adaptation (Sarason et al. 1983). In high‐risk environments, the quality and accessibility of social support networks may be constrained, which further challenges individual resilience (Kaniasty 2020). Ineffective or passive coping strategies, such as avoidance, may increase both individual and collective vulnerability by preventing proactive risk management (López‐Vázquez and Marván 2003; Lavanco 2003). Conversely, effective coping strategies, such as seeking social support, can foster resilience and enhance community preparedness (Kaniasty and Norris 2000). Therefore, a similar effect is likely within the Campi Flegrei context.

## 4 The Present Study

The Campi Flegrei area, with its complex history of bradyseism, emergency plans, and institutional communication failures, represents an example of how environmental stressors interact with psychosocial variables to shape individual and community well‐being. In this context, residents' capacity to flourish may be undermined by a living environment marked by alert levels, evacuation drills, perceived uncertainty, and ambiguous institutional messaging. Understanding well‐being as a dynamic, multidimensional state may therefore allow for more accurate monitoring of population health and more effective intervention strategies.

This study aims to explore the associations between specific psychosocial variables and the psychological impact of the ongoing emergency (impact of event) in the Campi Flegrei area. In the present manuscript, “impact of the event” refers to the psychological and emotional impact of the seismic and volcanic activity, operationalized through the Impact of Event Scale‐Revised (IES‐R). Specifically, the main objective is to assess how seismic and volcanic risk perception, perceived severity of volcanic phenomena, trust in institutions and media, and coping strategies influence this psychological impact. Furthermore, this research investigates mental well‐being within the context of the Campi Flegrei emergency using Keyes' MHC model. Specifically, we assessed the prevalence of flourishing and languishing in the Campi Flegrei population and examined whether the aforementioned variables differ across well‐being groups. Accordingly, the study is structured on two complementary levels of analysis.

First, at an individual level, we examine how specific psychosocial variables—risk perception, perceived severity, trust in institutions and media, and coping strategies—affect the impact of the event (Hypothesis 1). This level of analysis helps to identify the key mechanisms that shape people's psychological responses to risk and uncertainty.

Second, at a population level, we apply Keyes' categorical model to classify individuals as flourishing, moderately healthy, or languishing, and to explore whether these groups differ in the same psychosocial variables considered in H1 (Hypotheses 2a and 2b).

Thus, H1 and H2 are conceptually connected and mutually reinforcing: H1 identifies which variables are most influential in shaping the psychological impact of the emergency, whereas H2 describes how these variables differ across mental‐health groups, offering insights useful for targeted psychosocial interventions.

Taken together, these two levels of analysis reflect a process‐oriented and community‐based perspective consistent with contemporary disaster mental health research. Rather than focusing exclusively on psychological distress, the present study examines how chronic exposure to environmental threat shapes psychosocial functioning before the occurrence of a catastrophic event. In doing so, it clarifies how risk appraisal, trust, coping, and well‐being profiles become interconnected under prolonged conditions of uncertainty and collective threat.

This integrative approach is consistent with the rationale of Capone et al. (2020), who applied Keyes' framework in the Italian context during the COVID‐19 emergency and found that distinct mental‐health profiles were associated with different coping strategies and levels of perceived stress.

Starting from the above, in the present study, we tested the following hypotheses:

Hypothesis 1 (H1) The perception of risk, the perception of severity, and maladaptive coping strategies like denial positively predict the event impact, while trust in institutions and media and adaptive coping strategies like support‐seeking negatively predict the impact of the event.

Hypothesis 2a (H2a) It is expected that the categorical classifications of flourishing, moderately healthy, and languishing can be applied to the population residing in the Campi Flegrei area.

Hypothesis 2b (H2b) It is expected that the categorical classifications of psychological well‐being will distinguish levels of risk perception, coping strategies, trust in sources, and impact of the event among individuals classified as flourishing, moderately healthy, and languishing.

**Figure 1:**
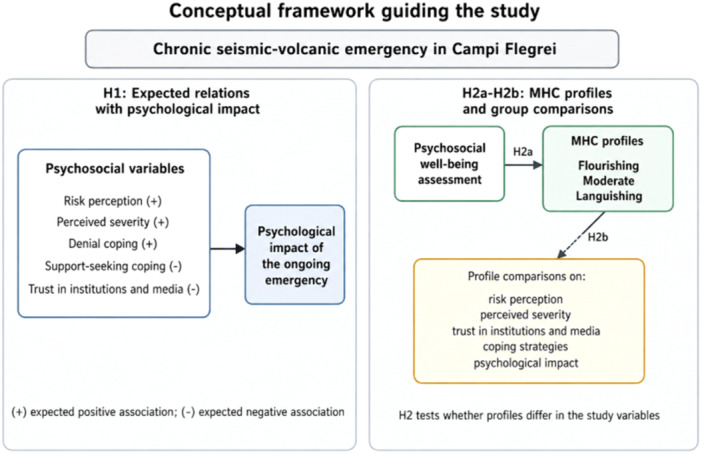
*Conceptual framework linking individual‐level and community‐level psychosocial variables to the psychological impact of the event (H1) and showing the use of Mental Health Continuum profiles for classification and group comparisons (H2a‐H2b)*.

To clarify this conceptual logic, Figure 1 summarizes the conceptual framework guiding the study. The figure distinguishes the H1 hypothesis, in which individual‐level variables and a community‐level variable are examined in relation to the impact of the event, from the H2 hypotheses, in which Mental Health Continuum profiles are used to classify participants and compare the same study variables across flourishing, moderate, and languishing groups.

The strengths of this research are clear. First, it addresses a significant gap in the literature by examining how a continuous seismic–volcanic threat shapes psychological well‐being in a highly exposed population before a disastrous event occurs. Few studies provide a detailed account of how residents living with protracted unrest actually experience daily life, perceive risk, trust institutions and media, and mobilize coping strategies in this phase; our findings offer a deeper, empirically grounded picture of those everyday experiences and psychological reactions. These data furnish concrete evidence that can inform both future research and risk‐management policies, highlighting the specific psychological challenges faced by residents and the need for targeted interventions to support mental health in place. Second, this study enriches the literature by applying Keyes's Mental Health Continuum (MHC) to a chronicemergency‐risk context, extending its use beyond the predominant applications in pandemic or general‐population research (e.g., Capone et al. 2020). Treating the MHC as an interpretive lens allows us to identify profile‐specific priorities (flourishing, moderate, languishing): for example, a single risk‐communication campaign is unlikely to be equally effective for all, whereas messages and supports calibrated to specific MHC profiles—such as strengthening self‐efficacy and plan clarity where trust is low, or bolstering social support and help‐seeking where denial coping is prevalent—are more likely to improve both impact‐of‐event outcomes and overall well‐being. In short, by linking risk perception, trust, and coping to population‐level well‐being profiles under chronic threat, the study turns a theoretical framework into actionable guidance for preparedness and communication in communities that live with persistent hazard.

Importantly, although the present design is cross‐sectional and correlational, this type of observational approach is particularly valuable in rapidly evolving emergency contexts such as Campi Flegrei, where psychosocial responses may fluctuate according to the intensity of ongoing seismic activity, public communication, and collective uncertainty. The present findings therefore provide a timely “snapshot” of psychosocial functioning during a particularly intense phase of chronic bradyseismic unrest.

## 5 Materials and Methods

### 5.1 Participants and Procedure

In this study, 602 participants residing in the red zone of the Campi Flegrei, a high‐risk volcanic area located in the western part of Naples and its surrounding municipalities, were involved. Of these, 532 were included in the final analysis. The remaining participants were excluded due to incomplete or invalid responses. Specifically, the excluded participants had failed to adequately respond to the “attention check” questions included in the survey, which were designed to ensure the accuracy and validity of the responses. The average age of the participants was 42.9 years (SD = 14.1). Of the total participants, 71.2% were women. Participants reported living in the following areas: 38% in the neighborhoods of Naples included in the red zone, 37% in Pozzuoli, 13% in Bacoli, 4% in Quarto, 3% in Monte di Procida, 1% in Marano, and 4% in Giugliano. The majority (98.6%) reported not living alone. 80.3% of participants had a medium‐to‐high level of education. 16.4% reported living with individuals with mobility impairments, and 19.2% reported living with elderly individuals. A noteworthy finding is that 49.4% of participants expressed an intention to relocate but stated they could not do so due to economic reasons, work commitments, or family obligations. A further 5.5% plan to move in the short term, while 11.1% intend to move in the long term.

Data were collected between March and June 2024 using an online questionnaire on the Google Forms platform. Participants were recruited primarily through social media, particularly Facebook community groups dedicated to residents of the municipalities located within the Campi Flegrei red zone. These online communities, which are highly active in sharing local news and information about the volcanic situation, allowed us to reach individuals living directly in the high‐risk areas. A snowball sampling technique was also employed, whereby participants were encouraged to share the survey link with friends, relatives, and acquaintances residing in the same municipalities. This strategy facilitated the inclusion of participants from different neighborhoods^1^ and social networks within the red zone, ensuring broader geographic and demographic coverage of the population at risk. At the same time, the use of social media recruitment and snowball sampling may have favored the participation of individuals who were more engaged with local risk‐related information or more motivated to participate, a consideration that should be taken into account when interpreting sample representativeness (Bethlehem 2010; Wright 2005). Interested individuals were able to access the online questionnaire through the provided link. The initial section of the questionnaire included information about the procedure, risks and benefits, privacy, informed consent, and the use of personal data, with the option to discontinue participation at any time. The average time to complete the survey was 25 min. This study was conducted following receipt of ethical approval from the local committee.

### 5.2 Measures

Perception of seismic and volcanic risk was assessed using the Ricci et al. (2013) scale, which consists of 4 items (α = 0.66). Consistent with the operationalization adopted by Ricci et al. (2013) and Barberi et al. (2008), this measure encompasses judgments of the likelihood and anticipated severity of a possible eruption, together with concern about its occurrence. The four items were combined to capture participants' overall appraisal of the risk associated with a possible volcanic eruption. Specifically, participants were asked to assess: the likelihood that a future volcanic eruption could affect their city (“How likely do you think it is that a volcanic eruption could affect your city?”), on a 5‐point Likert scale ranging from 1 (“absolutely improbable”) to 5 (“extremely probable”); the perceived severity of the consequences of an eruption, both for their community (“If a volcanic eruption were to occur, how severe do you think the consequences would be for your community?”) and for themselves and their family (“If a volcanic eruption were to occur, how severe do you think the consequences would be for you and your family?”), by using a 5‐point Likert scale ranging from 1 (“not severe at all”) to 5 (“extremely severe”); their level of concern about the possibility of an eruption (“How concerned are you about the possibility of a volcanic eruption?”), on a 5‐point Likert scale ranging from 1 (“not concerned at all”) to 5 (“extremely concerned”). Higher scores indicated greater risk perception.

Perception of severity was measured using a 7‐item scale (Ricci et al. 2013) (α = 0.86). The items assess the extent to which participants perceive various volcanic phenomena (earthquakes, volcanic eruption, ash fall, mudslides and debris, pyroclastic flows, tsunamis, ground subsidence/deformation/elevation) as a serious threat to their community. Ratings were made on a 5‐point scale from 1 (“not severe at all”) to 5 (“extremely severe”), with lower numbers indicating less severe consequences. The Perceived Severity scale is intended to capture comparative judgments of severity across hazard types (i.e., which phenomena respondents consider most damaging), rather than solely measuring the perceived likelihood or worry about a single eruption scenario.

Impact of Event (Stress from natural disaster threats) was measured using the Impact of Events Scale‐Revised (IES‐R) (Weiss 2007; Weiss and Marmar 1996) (α = 0.96). This self‐administered tool is used to assess the emotional impact of an event. The scale includes 22 items and can be used with both clinical and non‐clinical adult populations, evaluating the impact of events of varying severity. In the present study, the term *“*impact of the event*”* refers specifically to the psychological and emotional impact of the seismic and volcanic activity in the Campi Flegrei area, which represents the ongoing emergency situation under investigation. Therefore, the IES‐R total score operationalizes the *impact of the event*, quantifying the level of post‐traumatic stress symptoms associated with this specific natural disaster context. The IES‐R has three subscales: the Avoidance Subscale (e.g., “I tried to avoid being upset when I thought about it or when it was brought to my attention”), the Intrusiveness Subscale (e.g., “Anything that reminded me of it caused me to feel emotions related to it”), and the Hyperarousal Subscale (e.g., “I felt irritable and angry”). Before completing the questionnaire, participants were invited to briefly describe the traumatic event they had experienced in the past month, explicitly referring to seismic and volcanic activity in the Campi Flegrei area. These open‐ended descriptions, inherent to the scale's administration procedure, were not subjected to systematic qualitative coding; rather, selected excerpts were retained as descriptive material to ecologically contextualize participants' self‐reported psychological responses. For each item, participants responded in relation to this event, indicating how distressing each statement had been for them during the previous month^2^. Responses were given on a 5‐point Likert scale, ranging from 0 (“not at all”) to 4 (“extremely”). Scores ranged from 0 to 88. The presence of clinically relevant post‐traumatic stress symptoms was determined if the total score exceeded 33/88. The IES‐R has demonstrated strong psychometric properties across different populations and is considered a reliable and valid measure for assessing trauma‐related distress in individuals exposed to natural disasters.

Trust in institutions and media was assessed using seven items, adapting the scales from Trent et al. (2022) and Ricci et al. (2013). Specifically, four items measured trust in the ability of institutions to manage the emergency situation (e.g., “How much do you trust the decisions of your local municipality regarding the management of the Campi Flegrei emergency?”, “How much do you trust the decisions of the regional government regarding the management of the Campi Flegrei emergency?”, “How much do you trust the decisions of the national government regarding the management of the Campi Flegrei emergency?”, “How much do you trust the decisions of international political organizations regarding the management of the Campi Flegrei emergency?”), and three items assessed trust in civil protection, scientists, and the media (e.g., “How prepared do you think civil protection is to handle a potential eruption?”, “How much do you trust scientists to accurately predict a potential future eruption?”, “How much do you trust the media to report accurate information about a potential future eruption?”). All items were rated on a 5‐point scale from 1 (“not at all”) to 5 (“extremely”). (α = 0.87).

Coping strategies were measured using the Italian version of the Echelle Toulousaine de Coping (Depolo and Guglielmi 2000). It consists of four subscales measuring the following dimensions of coping: Control, Social Support, Avoidance, and Denial. Three items were selected for each dimension. For each item, the participant was asked to indicate the frequency with which they engage in the specific behavior on a scale from 1 (“never”) to 5 (“very often”). For the Control dimension, the following items were selected: “I analyze the situation to understand it better,” “I face the situation,” and “I control my emotions” (Cronbach's Alpha = 0.46). For the Social Support dimension, the following items were selected: “I seek solidarity and encouragement from others,” “I seek the help of my friends to calm my anxiety,” and “I feel the need to share what I am feeling with those close to me” (Cronbach's Alpha = 0.79). For the Avoidance dimension, the following items were selected: “I try to think about something else at all costs,” “I behave as if the problem does not exist,” and “I do other activities to distract myself” (Cronbach's Alpha = 0.49). Finally, for the Denial dimension, the following items were selected: “I isolate myself from others,” “I avoid meeting people,” and “When difficulties arise, I stop feeling anything” (α = 0.66). Given the very low internal consistency of the Control and Avoidance sub‐scales (α well below conventional thresholds of ~ 0.70), these two dimensions were excluded from all further analyses. The Denial sub‐scale (α = 0.66) falls into a marginal reliability range; while this does not meet the stricter α ≥ 0.70 criterion often cited, we retained this sub‐scale with caution, noting explicitly that results involving it should be interpreted tentatively. Indeed, prior research with brief coping inventories has sometimes proceeded with sub‐scales whose Cronbach's α ranged around 0.60–0.70 in analogous contexts (Addison et al. 2007).

Psychological well‐being was measured using the Mental Health Continuum‐Short Form (MHC‐SF) that consists of 14 items on 6‐point scales, ranging from 0 (“never”) to 5 (“every day”). (α = 0.92). It measures the degree of emotional well‐being (e.g., of item: “During the past month, how often did you feel happy”), social well‐being (e.g., of item: “During the past month, how often did you feel that you belonged to a community), and psychological well‐being (e.g., of item: “During the past month, how often did you feel that you had warm and trusting relationships with others"). In addition to providing a continuous measure of well‐being, the MHC‐SF allows for a categorical classification of mental health status following the Mental Health Continuum model. This classification is achieved by analyzing the frequency with which an individual experiences each of the 14 symptoms. Specifically, individuals who report experiencing at least seven symptoms “every day” or “almost every day,” including at least one symptom from the emotional well‐being dimension (e.g., feeling happy, interested in life, or satisfied), are classified as flourishing, indicating complete mental well‐being. Conversely, those who report experiencing at least seven symptoms “never” or “only once or twice,” again including at least one symptom of emotional well‐being, are classified as languishing, indicative of incomplete mental health. Participants not meeting the criteria for either flourishing or languishing are considered to have moderate mental health.

Finally, participants were also asked to report general demographic information such as gender and age, number and age of children, marital status, number of people they live with, financial resources, occupation, and educational background. Participants were also asked if they lived with elderly people or individuals with reduced mobility, and whether, considering the Campi Flegrei emergency, they had ever considered the possibility of relocating.

### 5.3 Statistical Analyses

Statistical analyses were conducted using SPSS version 29. Descriptive statistics were calculated for all studied variables, and associations between them were examined using Pearson's correlation coefficients (*r*). Linear regression analyses were performed to determine the predictors of the impact of the event. Finally, ANOVAs were conducted to examine whether there were significant differences in the investigated variables across different psychological well‐being groups (flourishing, moderately healthy, and languishing). Where significant differences were found, Bonferroni post hoc tests were performed to identify differences between specific groups in the means of the examined variables.

## 6 Results

### 6.1 Descriptive Analysis and Correlations Between Variables

Descriptive statistics and correlations between the considered variables are presented in Table 1. Frequency distributions for risk perception and the various information sources (e.g., trust in local and national institutions, media, and scientific sources) are detailed in Tables 2 and 3. Although trust was conceptualized and analyzed as a single construct due to its internal consistency, Table 3 also provides descriptive data for each specific source of authority and information. These data indicate meaningful differences in participants' confidence levels across local, regional, national, and international authorities, as well as in relation to scientists and the media, highlighting that citizens distinguish between institutional and scientific actors when forming judgments of trust. Additionally, a high score on the IES‐R scale was observed in 53.6% of participants, suggesting a potential presence of post‐traumatic symptoms, as their scores exceeded the scale's cut‐off (33/88). Beyond the quantitative data, personal testimonies were also collected from participants. Indeed, the IES‐R scale included a prompt asking them to briefly describe a traumatic event they experienced due to the seismic and volcanic activity in the Campi Flegrei region over the past month. Participants' open‐ended responses included references to sleep difficulties, hypervigilance, concerns for family members, anxiety, panic attacks, uncertainty about how to respond, and fears of something more severe occurring, including the loss of everything. Additional responses referred to resignation, habituation to repeated earthquakes, or a perceived sense of calmness and preparedness. Selected illustrative examples of these contents are considered in the Discussion section to contextualize the quantitative findings. These testimonies provide a glimpse into the individual experiences of participants and their psychological implications.

**Table 1:** Descriptive analyses and Correlations among the considered variables.

*N* = *532*	*Mean*	SD	*Range*	*1*	*2*	*3*	*4*	*5*	*6*	*7*
1. Risk perception	3.50	.68	1–5	1						
2. Perceived severity	3.90	.77	1–5	0.43**	1					
3. Impact of the event	35.02	22.36	1–88	0.41**	0.32**	1				
4. Trust in institutions and media	1.90	.66	1–5	−0.25**	−0.09*	−0.23**	1			
5. Denial coping	2.0	.89	1–5	0.16**	0.14**	0.34**	−0.11**	1		
6. Support‐seeking coping	2.93	1.04	1–5	0.13**	0.11**	0.32**	−0.01	0.12**	1	
7. Well‐being	3.29	1.05	1–6	−0.13**	−0.05	−0.20**	0.18**	−0.39*	0.06	1

* *p* < 0.05;

** *p* < 0.01.

**Table 2:** Likelihood, severity, and concern regarding future eruptions.

	Not at all *n* (%)	Slightly *n* (%)	Moderately *n* (%)	Very *n* (%)	Extremely *n* (%)
How likely do you think it is that a volcanic eruption could affect your city?	27 (5.1%)	193 (36.3%)	214 (40.2%)	70 (13.2%)	28 (5.3%)
If a volcanic eruption were to occur, how severe do you think the consequences would be for your community?	2 (0.4%)	16 (3.0%)	143 (26.9%)	120 (22.6%)	251 (47.2%)
If a volcanic eruption were to occur, how severe do you think the consequences would be for you and your family?	3 (0.6%)	22 (4.1%)	152 (28.6%)	113 (21.2%)	242 (45.5%)
How concerned are you about the possibility of a volcanic eruption?	16 (3.0%)	163 (30.6%)	192 (36.1%)	106 (19.9%)	55 (10.3%)

**Table 3:** Trust in information sources.

	Not at all *n* (%)	Slightly *n* (%)	Moderately *n* (%)	Very *n* (%)	Extremely *n* (%)
How much do you trust the decisions of your local municipality regarding the management of the Campi Flegrei emergency?	258 (48.5%)	195 (36.7%)	58 (10.9%)	15 (2.8%)	6 (1.1%)
How much do you trust the decisions of the regional government regarding the management of the Campi Flegrei emergency?	278 (52.3%)	178 (33.5%)	59 (11.1%)	14 (2.6%)	3 (0.6%)
How much do you trust the decisions of the national government regarding the management of the Campi Flegrei emergency?	229 (43.0%)	206 (38.7%)	79 (14.8%)	14 (2.6%)	4 (0.8%)
How much do you trust the decisions of international political organizations regarding the management of the Campi Flegrei emergency?	214 (40.2%)	206 (38.7%)	93 (17.5%)	15(2.8%)	4 (0.8%)
How prepared do you think civil protection is to handle a potential eruption?	180 (33.8%)	236 (44.4%)	85 (16%)	22 (4.1%)	9 (1.7%)
How much do you trust scientists to accurately predict a potential future eruption?	81 (15.2%)	187 (35.2%)	160 (30.1%)	76 (14.3%)	28 (5.3%)
How much do you trust the media to report accurate information about a potential future eruption?	259 (48.7%)	191 (35.9%)	62 (11.7%)	13 (2.4%)	7 (1.3%)

Finally, for exploratory purposes, ANOVA tests were conducted to examine whether there were differences in risk perception based on participants' municipality of residence. However, none of the analyses revealed significant differences, with all *p* > 0.05.

### 6.2 Regression Analysis: Predictors of Impact of Event

To test H1 and examine the role of risk perception, perceived severity, denial and support‐seeking coping strategies, and trust in institutions and media on the impact of the event, a linear regression analysis was conducted. The results (Table 4) indicated that risk perception, perceived severity, denial coping, and support‐seeking coping significantly and positively predicted the impact of the event, whereas trust in institutions and media negatively predicted it. Thus, H1 was partially confirmed, as contrary to expectations, both denial and support‐seeking coping strategies positively predicted the impact of the event.

**Table 4:** Regression analysis.

	B	*SE*	β	95% CI		*p*
				*LL*	*UL*	
Risk perception	8.454	1.349	0.26	5.804	11.103	0.000
Perceived severity	3.886	1.154	0.13	1.619	6.153	0.001
Trust in institutions and media	−4.184	1.244	−0.12	−6.627	−1.740	0.001
Denial coping	5.986	0.918	0.24	4.182	7.789	0.000
Support‐seeking coping	5.171	0.779	0.24	3.641	6.702	0.000
Fit of the model	Adj. R^2^ = 0.33 F (5, 526) = 52.753, *p* < 0.001					

### 6.3 Assessment of the Italian MHC–SF Among Campi Flegrei Residents

The categorical classification using the MHC–SF by Keyes (Petrillo et al. 2015) was applied to the data to assess the prevalence of mental well‐being categories among residents of the Campi Flegrei area. Using the MHC‐SF categorical criteria, the data reveal that 25.4% of the sample were languishing (*N* = 135), 51.9% were moderately mentally healthy (*N* = 276), and 22.7% were flourishing (*N* = 121).

One‐way ANOVAs were conducted to examine whether the study variables differed across MHC‐SF categories. As reported in Table 5, significant group differences emerged for risk perception, perceived severity, impact of event, trust in institutions and media, and denial coping, whereas support‐seeking coping did not differ significantly across groups. Bonferroni post hoc comparisons showed that languishing participants generally reported a more vulnerable psychosocial profile, with higher risk perception, perceived severity, impact of event, and denial coping than one or both of the other groups. Trust in institutions and media was lower among languishing participants than among moderate and flourishing participants. Thus, H2b was partially supported.

**Table 5:** Psychosocial variables by MHC‐SF category.

Variable	Category	M (SD)	F	*η* ^2^	Sig. pairwise comparisons
Risk perception	Languishing	3.65 (0.69)	4.29*	0.16	L > M*
	Moderate	3.45 (0.65)			
	Flourishing	3.45 (0.70)			
Perceived severity	Languishing	4.03 (0.79)	3.97*	0.02	L > M*
	Moderate	3.81 (0.74)			
	Flourishing	3.94 (0.79)			
Impact of the event	Languishing	41.91 (22.87)	9.22***	0.03	L > M***; F < L***
	Moderate	33.28 (21.07)			
	Flourishing	31.28 (23.16)			
Trust in institutions and media	Languishing	1.67 (0.58)	10.95***	0.04	L < M***; F > L***
	Moderate	1.98 (0.66)			
	Flourishing	1.97 (0.70)			
Denial coping	Languishing	2.46 (0.96)	43.61***	0.14	L > M***; M > F***; F < L***
	Moderate	1.98 (0.86)			
	Flourishing	1.50 (0.53)			
Support‐seeking coping	Languishing	2.87 (1.09)	0.36	0.00	
	Moderate	2.96 (0.97)			
	Flourishing	2.96 (1.13)			

*Note:* F = Flourishing; M = Moderate; L = Languishing.

* *p* < 0.05

*** *p* < 0.001.

These analyses emphasize that individuals classified as languishing generally exhibit higher levels of adverse psychological outcomes compared to their moderate and flourishing counterparts.

## 7 Discussion

This study explored the psychological responses of residents in the Campi Flegrei area to seismic and volcanic activity by examining the role of specific psychosocial variables (i.e., risk perception, perceived severity, coping strategies, trust in institutions and media) in shaping the psychological impact of the event. Consistent with our aims, the discussion emphasizes an in‐depth picture of the general psychological processes involved in living under chronic threat and the distinct mental‐health profiles observed in the sample, clarifying how these elements co‐occur in everyday adaptation to the ongoing emergency.

This topic is of critical importance because natural phenomena such as ground deformation (bradyseism) and constant volcanic activity in this area represent a real and persistent threat with significant material and psychological consequences for the local population (Galderisi and Limongi 2024).

The potential presence of post‐traumatic symptoms (reported by 53.6% of participants) is particularly concerning, indicating that the local population experiences significant psychological distress, not necessarily due to the direct experience of a catastrophic event, but rather due to prolonged and uncertain anticipation of a potentially devastating occurrence. Residents have experienced a series of seismic and volcanic events that occur with a certain frequency and intensity in the region, though not always catastrophic. As the literature suggests, repeated exposure to threats can accumulate a level of stress that exceeds coping capacities, potentially leading to the development of PTSD symptoms (Yehuda 2002). In line with a process‐oriented reading, these findings may be indicative of anticipatory stress and sustained threat appraisal under repeated swarms and alerts, rather than post‐event symptomatology. Moreover, nearly half of the participants expressed a desire to leave the area but were hindered by external constraints, such as limited economic or social resources. This underscores that decisions about personal safety are influenced not only by risk perception but also by the availability of resources, as outlined in the Conservation of Resources (COR) model (Hobfoll 1989).

The findings revealed that both risk perception and perceived severity were particularly high among participants, confirming a widespread awareness of the danger. This result is consistent with previous research on risk perception in natural threat contexts, where prolonged exposure to perceived risk elevates levels of concern and psychological activation (Slovic 1987). In contrast, trust in institutions and media was found to be extremely low, reflecting a generalized distrust in authorities, scientists, and the media. This lack of trust appears to be associated with heightened perceptions of vulnerability. Consistent with these findings, the study by Galderisi and Limongi (2024) also identified a marked awareness of volcanic risk and a heightened sense of concern among residents of the Campi Flegrei area. Their research highlights a persistent lack of trust in institutional authorities, despite the considerable efforts made to enhance public preparedness and disseminate risk‐related information. Notably, while awareness and preparedness have increased over time, confidence in both the effectiveness of emergency plans and the capacity of authorities to manage potential crises remains limited. This suggests that institutional distrust may not merely reflect a lack of information but rather deeper concerns regarding the clarity, accessibility, and perceived reliability of official risk communication strategies. Additionally, the data analyses did not reveal significant differences in risk perception between residents of different municipalities. This lack of differences might be explained by the fact that the study sample was drawn entirely from the so‐called “red zone,” the area with the highest risk of eruption. In this context, it is understandable that, despite residing in different municipalities, participants share a similar risk perception due to their common exposure to the threat. The “red zone” is constantly monitored and subject to evacuation plans (Istituto Nazionale di Geofisica e Vulcanologia 2024), which may foster a collective awareness of vulnerability regardless of the specific municipality of residence. Future research might explore whether differences in risk perception exist between those living inside and outside the red zone, in order to better understand how proximity to the threat influences behavior and preparedness.

The results of the regression analyses partially confirm H1, highlighting the central role of risk perception, perceived severity, and maladaptive coping strategies in predicting event impact. In particular, high‐risk perception and perceived severity are significantly associated with greater psychological impact, suggesting that higher perceived threat is associated with greater psychological burden. In fact, risk perception emerged as the strongest predictor of the perceived impact of the event. This finding aligns with previous research, such as Lopez Vazquez (2001), who demonstrated that individuals' perception of risk was directly linked to their psychological stress levels in populations exposed to natural disasters. Similarly, Norris et al. (2002) found that individuals with higher levels of risk perception reported greater psychological distress, including symptoms of stress and trauma. Furthermore, Ionescu et al. (2021) identified risk perception as the primary determinant of emotional distress in the aftermath of seismic events, further supporting the critical role that perceived risk plays in shaping psychological outcomes in crisis situations.

On the other hand, the low level of trust in local, national, and international institutions, as well as in scientists and media, was negatively associated with the impact of the event. Although this pattern may be compatible with a potentially protective role of trust, the cross‐sectional design does not allow us to determine the direction of the association or the mechanisms underlying it. Previous studies have shown that high confidence in authorities and scientific information reduces anxiety and enhances preparedness in the face of natural emergencies. Conversely, a lack of trust contributes to a greater sense of vulnerability, as individuals do not feel they have adequate control or balanced management of the situation (Terpstra 2011). However, this relation can also be understood through frameworks of trust erosion in contexts of chronic threat. Institutional trust is dynamic and contingent upon perceptions of competence, transparency, responsiveness, and value alignment of authorities (Peters et al. 1997). In situations characterized by prolonged and uncertain hazards, such as the Campi Flegrei bradyseismic crisis, institutional trust may gradually weaken if communication is perceived as inconsistent, insufficiently transparent, or unable to reduce uncertainty. Previous research suggests that diminished institutional trust may heighten perceptions of vulnerability and reduce confidence in mitigation measures, thereby influencing how individuals appraise and emotionally respond to risk (Terpstra 2011; Stretesky et al. 2023). From this perspective, the negative association observed between institutional/media trust and the impact of the event in the present study may be compatible with emerging processes of trust erosion in chronically exposed communities. At the same time, given the cross‐sectional nature of the present study, this interpretation remains necessarily tentative and future longitudinal research may help clarify whether trust erosion develops over time in response to repeated bradyseismic activity and how such dynamics shape both psychological adaptation and preparedness behaviors in exposed populations.

Although media exposure was not directly assessed in the present study, it may represent an important factor to consider in future research on chronic environmental threat. Previous evidence suggests that repeated exposure to risk‐related information can shape emotional and behavioral responses, with fear potentially mediating the relationship between information exposure and protective behavior (Scopelliti et al. 2021). In contexts such as Campi Flegrei, future studies may further explore how media framing and prolonged exposure to risk communication influence trust, perceived threat, and psychological adaptation over time.

Regarding coping strategies, the results revealed a complex pattern. While both maladaptive strategies, such as denial, and adaptive strategies, such as support‐seeking, were commonly reported, their role in managing distress appears varied. Many participants engaged in both types of coping, reflecting the ongoing challenge of dealing with prolonged uncertainty and perceived threat. At the same time, these findings should be interpreted with caution due to several psychometric limitations concerning the coping measures employed. Specifically, the Control and Avoidance sub‐scales were excluded from the analyses due to unacceptably low internal consistency (Cronbach's α = 0.46 and 0.49, respectively), thereby reducing the breadth of the coping construct examined in the present study. The retained Denial sub‐scale achieved a marginal reliability coefficient (α = 0.66), which, although below the conventional threshold of 0.70, falls within a range that has occasionally been considered acceptable in coping research when interpreted cautiously (e.g., Addison et al. 2007).

To further contextualize this result, qualitative testimonies collected through the IES‐R prompt provide additional insight into how some residents psychologically managed prolonged exposure to uncertainty. Although many narratives reflected fear, hypervigilance, and emotional distress, some participants appeared to describe forms of emotional distancing, resignation, or normalization of the threat that may be broadly compatible with defensive coping processes. For example, one participant reported feeling “almost resigned” to the situation (“quasi rassegnato”), while another stated that they had “become used to the earthquakes” (“ho fatto ormai l'abitudine alle scosse”). Similarly, some residents described themselves as relatively calmer and more prepared because of repeated exposure (“noi della zona siamo più calmi e preparati”), suggesting possible attempts to preserve psychological functioning despite persistent threat exposure. While these accounts cannot be interpreted as direct indicators of denial coping, they offer contextual support for the idea that some residents may engage in forms of emotional regulation or psychological distancing to manage chronic uncertainty.

Moreover, the data confirms their influence on the psychological impact of the event. Maladaptive coping, specifically denial, was positively correlated with event impact, in line with H1. This suggests that denying or avoiding the perceived threat not only fails to alleviate stress but may even exacerbate it, as confirmed by studies highlighting the limited effectiveness of avoidant coping in risky situations (Lazarus and Folkman 1984). Conversely, social support–seeking coping, typically considered an adaptive strategy, also showed a positive correlation with event impact. Although initially counterintuitive, this unexpected finding may reflect the psychosocial dynamics of prolonged and collectively experienced threat. In contexts such as Campi Flegrei, where uncertainty is persistent and socially shared, seeking support may not necessarily mitigate distress but may instead expose individuals to repeated exchanges centered on fear, uncertainty, and catastrophic expectations, thereby contributing to the maintenance of emotional activation.

This interpretation is consistent with research on communal coping, which suggests that the social sharing of stressful experiences may have both protective and adverse consequences depending on how collective meanings surrounding the stressor are negotiated (Afifi et al. 2012). While shared communication can foster solidarity and emotional regulation, prolonged exposure to uncertainty may also foster processes of communal rumination, whereby repeated discussions surrounding the threat reinforce perceptions of danger and helplessness. Similarly, studies on emotional contagion indicate that fear and anxiety may spread through social networks in communities exposed to chronic uncertainty, amplifying vigilance and psychological burden rather than attenuating them (Páez et al. 2015).

The present findings may therefore point to a form of “social support paradox,” whereby support‐seeking remains highly activated precisely because distress is elevated, while simultaneously offering limited buffering effects in an environment characterized by persistent collective stress. This interpretation is broadly consistent with evidence showing that social support may become less protective when exchanges occur within highly distressed environments or inadvertently reinforce worry rather than coping efficacy (Bolger et al. 2000; Maisel and Gable 2009).

Overall, these results suggest that coping processes in high‐risk communities differ qualitatively from those observed in isolated stress situations. Under conditions of prolonged and collectively experienced uncertainty, coping appears to function as a dynamic and context‐dependent process, shaped not only by individual resources but also by the broader social environment in which threat is experienced and negotiated. Future research should therefore employ broader coping measures and longitudinal designs to better capture the evolution of coping responses in populations exposed to persistent environmental risk.

Consistent with our expectations, the results suggest that residents exhibit a modest level of well‐being. Given the persistent exposure to natural risks and the ongoing emergency conditions in the area, this outcome further emphasizes the adverse psychological effects of prolonged exposure to such threats. In line with H2a and H2b, the findings further support the applicability of Keyes (2007) Mental Health Continuum framework in a context of chronic environmental threat, highlighting meaningful psychosocial differences across flourishing, moderately healthy, and languishing individuals. More in detail, flourishing individuals function better than moderately healthy individuals, who in turn function better than languishing individuals in terms of various psychosocial assets (Keyes 2002). Flourishing is associated with desirable outcomes such as high trust in institutions and media. Specifically, risk perception was significantly higher in the languishing group compared to the moderate group, suggesting that compromised mental well‐being is linked to greater sensitivity and concern regarding the threat. A similar pattern emerged for perceived severity, where languishing individuals reported higher levels than their moderate counterparts, confirming that individuals with more fragile psychological states tend to evaluate threats as more severe. With regard to the impact of the event, significant differences were observed across all three groups: languishing individuals showed the highest levels, followed by moderately healthy individuals, while flourishing individuals reported the lowest levels. This pattern indicates that levels of psychological impact differed across the MHC‐SF well‐being groups, with languishing participants reporting greater impact than the other groups. Notably, trust in institutions and media was highest among flourishing individuals, followed by moderate individuals, and lowest among languishing individuals. This further highlights that a higher degree of trust in institutions and media may promote psychological well‐being by reducing anxiety and uncertainty regarding threat management (Terpstra 2011). Additionally, negative coping strategies, such as denial, were more frequently employed by languishing individuals compared to the moderately healthy and flourishing groups. These findings confirm the link between mental health and the emotional impact of a persistent environmental threat.

Overall, these results indicate that individuals with lower overall mental well‐being (i.e., those in the languishing group) are particularly vulnerable to negative psychological outcomes when facing natural phenomena. The elevated risk perception and impact of the event, combined with a greater reliance on maladaptive coping strategies, suggest a maladaptive response pattern that may exacerbate distress. Conversely, higher levels of trust in institutions and media among flourishing individuals might serve as a buffer, potentially reducing both the perceived severity and the impact of the event. These findings confirm that Keyes' Mental Health Continuum is applicable to the Campi Flegrei context.

However, it is important to acknowledge the study's limitations. The sample was of convenience, which may affect the generalizability of the findings, as participants might not fully represent the broader variability within the Campi Flegrei population. More specifically, participants were recruited primarily through Facebook community groups and snowball sampling, a strategy that may have introduced selection bias. Online and self‐selected recruitment procedures may disproportionately attract individuals who are more engaged with the topic under investigation or more motivated to participate, potentially limiting sample representativeness (Bethlehem 2010; Wright 2005). In the present case, it is possible that residents more concerned about or emotionally involved in the ongoing seismic–volcanic unrest were more likely to participate, which may have contributed to the relatively high prevalence of post‐traumatic symptoms observed in the sample. Accordingly, these findings should be generalized with caution. Additionally, the self‐report measures employed are susceptible to biases such as social desirability, potentially influencing responses, especially in a context as sensitive as risk and negative emotion assessment. As previously mentioned, another limitation concerns the internal consistency of some coping subscales, which showed suboptimal reliability coefficients. Although this issue was addressed in the analytical strategy through the exclusion of subscales with very low reliability, it nevertheless highlights the need for further psychometric refinement of coping measures when applied in high‐risk community contexts. In particular, future research should aim to validate more robust and context‐sensitive instruments capable of capturing coping processes under conditions of chronic environmental stress and uncertainty. Moreover, the cross‐sectional design of the study prevents any inference about the directionality or causality of the observed associations. At the same time, this design allowed us to capture residents' psychosocial functioning during a particularly intense phase of ongoing bradyseismic activity, providing a timely picture of how chronic environmental threat is experienced at the community level. In rapidly evolving emergency contexts, such observational “snapshots” may offer valuable insights for both theory development and immediate psychosocial intervention planning. Future research employing longitudinal or experimental designs could provide valuable insights into the temporal and causal dynamics underlying these associations and help clarify how psychological and social responses evolve over time under conditions of chronic environmental threat. Finally, the study did not account for other potentially influential variables, such as sense of community and self‐efficacy, which might have impacted the results.

Nonetheless, the strengths of this research are numerous and have been discussed in the preceding paragraphs. Here, we simply note that the findings deepen understanding of residents' daily experiences and psychological responses under persistent seismic–volcanic threat and provide actionable evidence to guide future research and risk‐management policies, underscoring the need for targeted mental‐health interventions. In sum, integrating psychological insights into emergency planning can strengthen community resilience: by addressing psychological and emotional as well as practical needs, interventions ensure that individuals, families, and the broader community are better equipped to withstand the ongoing challenges of life in a high‐risk volcanic area.

## 8 Conclusion

In conclusion, this research contributes to our understanding of individuals' responses in contexts of high natural risk by offering valuable insights for developing intervention strategies aimed at reducing psychological impact and promoting resilience in at‐risk areas such as the Campi Flegrei. Overall, the findings meet the study's aims by providing an in‐depth account of the general psychological processes associated with living under constant seismic–volcanic threat and by describing the different mental‐health profiles present in the exposed population. By clarifying how appraisal, trust, and coping relate to the reported impact of the event and vary across profiles, the study identifies concrete levers for intervention at individual, group, and community levels—relevant to Campi Flegrei and comparable high‐risk contexts. The analysis of residents in the red zone has emphasized the importance of targeted psychosocial interventions that consider not only individual characteristics but also community dynamics and the quality of information received. One key implication of this study is the need to enhance trust in institutions and media through more effective communication strategies. Risk communication should not be limited to the transmission of hazard information, but should provide clear, timely, consistent, and actionable messages that support preparedness and informed decision‐making (Reynolds and W. SEEGER 2005). Without trust in institutional and media sources, the public is more likely to disregard even accurate data and recommended safety measures (Chryssochoidis et al. 2009). In this sense, trust‐building should be understood as a sustained process, because trust in information sources can influence whether individuals translate risk information into protective action (Paton 2008).

Importantly, the present findings suggest that communication strategies in prolonged emergency contexts may benefit from being calibrated according to residents' psychosocial profiles rather than relying on a uniform approach. Targeted communication is particularly useful when population subgroups differ in relevant psychological characteristics, because it can increase the perceived relevance of messages (Kreuter and Wray 2003; Noar et al. 2007). In the present study, residents classified as languishing reported higher psychological impact of the event and perceived severity, together with lower trust in institutions and media. For this subgroup, local authorities could prioritize messages that are transparent, concrete, and action‐oriented: for example, repeated information on evacuation procedures, family preparedness planning, available support services, and specific protective actions that residents can realistically implement. Conversely, for individuals with moderate or flourishing profiles, who exhibit higher institutional trust and more adaptive coping resources, communication could shift from a primary focus on foundational preparedness and procedural guidance to active community engagement. For these subgroups, authorities might promote peer‐led preparedness initiatives or community leadership roles, leveraging their psychological robustness to foster collective resilience. This approach is consistent with social‐cognitive models of disaster preparedness, which emphasize the role of outcome expectancy, self‐efficacy, trust, and community participation in motivating protective behavior (Paton 2003, 2008).

The finding that support‐seeking coping positively predicted the impact of the event also has practical implications. Rather than simply encouraging generic social support, interventions should promote structured forms of support that combine emotional containment with practical problem‐solving. For example, community meetings, psychoeducational sessions, and facilitated preparedness activities could help residents share concerns while also receiving reliable information, identifying concrete actions, and being connected to available services. This is consistent with mental health and psychosocial support guidelines, which emphasize practical help, access to information, connection with social support, and community‐based resources as key components of support in emergency settings (Inter‐Agency Standing Committee 2007; World Health Organization, War Trauma Foundation, & World Vision International 2011).

While it may not always be possible to intervene in natural events, it is both feasible and necessary to reduce the vulnerability associated with socio‐cultural functions such as communication and prevention (Frudà 1997). The perspective of community psychology emphasizes that interventions in crisis contexts should address not only individual psychological responses but also the broader social context. Community‐based interventions aimed at enhancing social capital and improving communication between institutions and citizens are crucial to fostering resilience and ensuring better preparedness for future crises (Paton and Johnston 2001). Drawing from community psychology perspectives, interventions should strengthen not only social support networks but also collective efficacy and participatory engagement, empowering communities to respond more effectively to crisis situations (Rappaport 1987). Such approaches may strengthen both psychological well‐being and the broader social fabric, thereby increasing communities' capacity to adapt to future challenges.

Accordingly, the promotion of psychosocial health should not be limited to crisis intervention but should be understood as an ongoing process of prevention, preparedness, and adaptive response. Only through an integrated approach that actively promotes mental and social well‐being can resilience be strengthened among populations exposed to natural risks. Overall, our work broadens the knowledge base on living with persistent natural threats and underscores the importance of incorporating psychological perspectives into both the theory and practice of disaster risk management.

## Funding

The authors have nothing to report.

## Ethics Statement

All procedures performed in studies involving human participants were in accordance with the ethical standards of the Ethical Committee of Psychological Research of the Department of Humanities of the University of Naples Federico II and with the 1964 Helsinki declaration and its later amendments or comparable ethical standards.

## Consent

Informed consent was obtained from all individual participants included in the study.

## Conflicts of Interest

The authors declare that they have no known competing financial interests or personal relationships that could have appeared to influence the work reported in this paper.

## Data Availability

The data that support the findings of this study are available from the corresponding author upon reasonable request. Questionnaires and data are available on request to the corresponding author.

## Endnotes

1 The following inclusion criteria were applied: being of legal age and residing in one of the municipalities of the red zone of the Campi Flegrei, which includes Pozzuoli, Bacoli, Monte di Procida, Quarto, a portion of Marano, a limited area of Giugliano, and certain districts of Naples (i.e., Bagnoli, Fuorigrotta, Pianura, Soccavo, Posillipo, Chiaia, part of Arenella, Vomero, Chiaiano, and San Ferdinando).

2 Personal testimonies from participants were also collected. In the questionnaire administered, respondents were asked to briefly describe a traumatic event experienced as a result of the seismic and volcanic activity of the Campi Flegrei during the past month. Among the answers provided, several recurring themes emerged that deeply reflect the psychological impact of the situation. Many participants reported experiencing a range of significant difficulties. In particular, a large proportion of respondents reported severe sleep disturbances, often accompanied by a state of constant alertness. This condition of hypervigilance is characterized by persistent fear and heightened sensitivity to tremors and noises, which are interpreted as warning signs of a potentially imminent catastrophic event. The literature suggests that sleep disturbance is a frequent symptom of post‐traumatic stress disorder (PTSD), and such difficulties may be exacerbated by the perception of an imminent and ongoing threat (Rothbaum et al. 1992). Anxiety and sustained alertness can significantly reduce quality of life and undermine the psychological well‐being of residents.
